# Incidence and Risk Factors for Tuberculosis in People Living with HIV: Cohort from HIV Referral Health Centers in Recife, Brazil

**DOI:** 10.1371/journal.pone.0063916

**Published:** 2013-05-10

**Authors:** Joanna d’Arc Lyra Batista, Maria de Fátima Pessoa Militão de Albuquerque, Magda Maruza, Ricardo Arraes de Alencar Ximenes, Marcela Lopes Santos, Ulisses Ramos Montarroyos, Demócrito de Barros Miranda-Filho, Heloisa Ramos Lacerda, Laura Cunha Rodrigues

**Affiliations:** 1 Centro de Pesquisas Aggeu Magalhães/Fiocruz, Recife, Brazil; 2 Hospital Correia Picanço, Recife, Brazil; 3 Universidade Federal de Pernambuco, Recife, Brazil; 4 Universidade de Pernambuco, Recife, Brazil; 5 London School of Hygiene and Tropical Medicine, London, United Kingdom; St. Petersburg Pasteur Institute, Russian Federation

## Abstract

**Objective:**

To identify the incidence of and risk factors for tuberculosis in people living with HIV (PLHIV).

**Design:**

Observational, prospective cohort study.

**Methods:**

A total of 2069 HIV-infected patients was observed between July 2007 and December 2010. The Kaplan-Meier method was used to estimate the probability of survival free of tuberculosis, and Cox regression analysis to identify risk factors associated with the development of tuberculosis.

**Results:**

Survival free of tuberculosis (TB) was 91%. The incidence rate of tuberculosis was 2.8 per 100 persons/years. Incidence of tuberculosis was higher when subjects had CD4 cell count <200 cells/mm^3^; were not on antiretroviral therapy; in those who had, a body mass index <18.5 kg/m^2^, anemia (or were not tested for it), were illiterate or referred previous tuberculosis treatment at entry into the cohort. Those not treated for latent TB infection had a much higher risk (HR = 7.9) of tuberculosis than those with a negative tuberculin skin test (TST). Having a TST≥5 mm but not being treated for latent TB infection increased the risk of incident tuberculosis even in those with a history of previous tuberculosis.

**Conclusions:**

Preventive actions to reduce the risk of TB in people living with HIV should include an appropriate HAART and treatment for latent TB infection in those with TST≥5 mm. The actions towards enabling rigorous implementation of treatment of latent TB infection and targeting of PLHIV drug users both at the individual and in public health level can reduce substantially the incidence of TB in PLHIV.

## Introduction

Tuberculosis (TB) is a major cause of morbidity and mortality in people living with HIV (PLHIV) [1]; it can accelerate the course of HIV infection and decrease the survival of patients with AIDS [2].TB mortality rates are significantly higher for PLHIV (RR = 9), and early detection reduces the chances of mortality [3], [4]. In 2008, WHO launched a strategy for tuberculosis control with three goals (the so-called three I’s): increased case detection, preventive therapy with isoniazid, and the control of HIV infection with large-scale supply of antiretroviral therapy [5].

Studies of the risks for developing tuberculosis in PLHIV have focused mainly on clinical and laboratory factors. Studies on socioeconomic factors and lifestyle habits of PLHIV who develop tuberculosis are scarce in spite of strong evidence of an association in the general population [6]. WHO in their recommendation for collaboration between TB/HIV control, recommends early identification of those at risk of developing TB to introduce early intervention.

This study aimed to estimate the incidence density and identify risk factors for TB in PLHIV receiving medical care at two referral centers for HIV infection in Recife, the capital city of Pernambuco, Brazil. Pernambuco is the Brazilian state with the second highest TB mortality rates (4.0 deaths per 100,000 inhabitants per year), almost double the national rate [7].

## Methods

### Study Design and Population

This is a prospective cohort study of PLHIV in routine care, with individuals receiving medical care in two referral hospitals (Oswaldo Cruz University Hospital and Correia Picanco Hospital), responsible for the care of about 70% of PLHIV in the state. Treatment for TB and for HIV is distributed free of charge by the health services, restricted to cases notified to the System of Notification of Infectious Diseases (SINAN)/Pernambuco (PE). Recruitment started in July 2007 and ended in June 2010. Follow up ended in December, 2010. All individuals aged ≥18 years consenting to participate and responding to a questionnaire were included. Since our objective was to understand the incidence of tuberculosis, patients already being treated for TB at the time of entry into the cohort, or who developed TB during the first month of follow-up, were excluded.

### Case Ascertainment and Definition

#### Incident TB was identified in three ways

(i) A new diagnosis of TB by the attending physician (according to the Ministry of Health of Brazil guidelines, that is based on clinical findings, direct investigation of Acid-fast bacillus –AFB -smear and culture for *M.tb* [8]). (ii) Cases of TB notified to the surveillance system (SINAN/PE) during the follow up period and ascertained through record linkage and (iii) deaths from TB in Mortality Information System (SIM/PE), ascertained through record linkage. All linkage used the probabilistic linkage program RecLinkIII [9].

### Predictor Variables

We investigated biological, socioeconomic, habits and lifestyle factors and variables related to HIV and TB. Alcohol consumption was defined based on reported number of drinks per day, according to the Centers for Disease Control and Prevention [10], which classifies people into abstainers, light drinkers (individuals who did not drink or drank up to two drinks per day for men, and one drink per day for women) and heavy drinkers (more than two drinks per day for men and more than one drink per day for women). Smoking was classified into three categories: non-smokers (individuals who had never smoked), former smokers (those who did not smoke when entering the study and had stopped smoking at least six months earlier) and smokers (those who smoked at the time of study or had stopped smoking less than six months earlier). For the multivariate Cox regression analysis, the categories ex-smokers and never smokers were grouped into a single category. Individuals who reported past or current use of marijuana, cocaine or crack were classified as drugs users.

Subjects were classified as having anemia, if the result of the entry blood test showed hemoglobin <12 g/dL in women and <14 g/dL in men, according to the EuroSIDA criterion [11]. As this test was not done in all patients, there were a considerable numbers of missing values (22.9%); those without a test result were treated as a third category.

CD4 cell count at baseline was categorized as ≥200 cells/mm^3^ or <200 cells/mm^3^. The use of antiretroviral drugs was measured at various points during the follow-up and at each point was categorized as using HAART or not. The tuberculin skin test (TST) was performed during the patients’ follow-up by the attending clinician using their own clinical judgment. After conducting the TST, a positive result was considered indication for treatment of latent tuberculosis infection (LTBI) as recommended by Brazilian Ministry of Health. The treatment of LTBI consists of isoniazid, 300 mg/day for six months [12]. Since many were not tested, and many with indication for treatment did not receive treatment, this variable was categorized into 4 levels, following Golub et al: not tested, tested negative (where treatment is not recommended), tested positive (with treatment recommended and done) and tested positive (treatment recommended but not done) [13].

After agreeing to participate and signing the informed consent, individuals were interviewed by a nursing technician who filled a questionnaire developed for this research; this was repeated during the follow up (ideally every six months) during their routine health care visits.

### Statistical Analysis

Follow up time for those developing tuberculosis was the time from start of follow-up to the date of tuberculosis diagnosis, defined as date of initiating TB treatment (confirmed by the notification form in the SINAN/PE) or date of death in those dying from tuberculosis before starting treatment (confirmed by the SIM/PE). Follow-up time for those not developing tuberculosis was the time from start of follow up until the date of death from other causes, date of transfer to another health service not in the study, or the date of the end of the study, 31 December 2010. Kaplan-Meier method was used to calculate the probability of survival free of tuberculosis, overall and stratified by study variables. The statistical significance of differences between the Kaplan-Meier (KM) curves was checked by log-rank test <0.05. Since use of HAART changed for many patients during the follow up, this was analyzed as time-dependent variable in the Cox regression model, allowing us to take into account changes in risk over time. To identify factors associated with incident TB, hazard ratios (HR) were calculated [14]. The proportional hazards assumption was tested in the bivariate and multivariate models. Significant variables (p≤0.20) in bivariate analysis were introduced one by one according to their statistical significance and clinical and epidemiological importance in the forward multivariate Cox regression model, where they remained if the p value was <0.05. We used Schoenfeld residuals (phtest) to test for the proportional-hazards assumption. As antiretroviral treatment did not respect the proportional-hazard assumption, the effect of the other variables in the multivariate model was stratified by HAART. Data were analyzed using the software Stata 11.2 (Stata-Corp LP, College Station, TX).

### Ethical Considerations

This study is part of the project which was approved by the Ethics Committee of Federal University of Pernambuco (CEP/CCS/UFPE 254/05).

## Results

The “Clinical-epidemiological study of co-infection tuberculosis/HIV in Recife” cohort consisted of 2362 PLHIV. Those who were receiving TB treatment at entry or during the first month of follow up (293–12.4%) were excluded in this analysis. The remaining 2069 patients were followed up for the study reported here: 147 (7.1%) patients developed TB; 144 were reported to SINAN/PE and the other three died before treatment started and had TB as the cause of death in the death certificate in SIM. Of the remaining 1922, 96 died and the death certificate registered at SIM did not mention TB, 8 transferred out and 1818 were alive and without TB at the end of the study.

**Figure 1:**
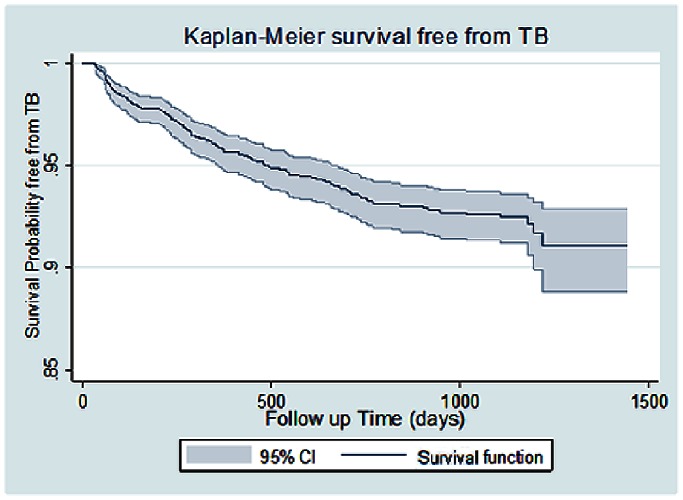
Kaplan-Meier probability of TB-free Survival.

The individual mean follow up time was just over 2.5 years (928.7 days). The total person-years at risk was 5259, and the incidence rate of TB was 2.8 per 100 person-years. Figure 1 shows the Kaplan-Meier probability of survival free of tuberculosis: 91% by the end of follow-up.

Tables 1 and 2 present the frequency distribution of the exposure variables. Demographic, socioeconomic and lifestyle factors are presented in table 1, and clinical/laboratory/treatment factors related to general health, to HIV, and to TB in table 2. The HR, 95% CI and the p-value for the bivariate association between each studied factor and incidence of TB; and the p-value for the proportional-hazards assumption. Most (61%) of the patients were male, 298 (14.5%) of the patients did not live in the metropolitan region of Recife, 11.2% were illiterate and 31.3% lived in families where the monthly income of the head of the family was less than one minimum wage (on average 170dollarsover the study period). Heavy drinking was reported by 11.4% of all patients; past or current use of marijuana, cocaine or crack was reported by 27.4% and most patients in the cohort (54.9%) were either smokers (29%) or ex-smokers (25.9%). Table 2 shows that a total of 1635 (79.3%) patients were already on antiretroviral therapy at entry into the cohort and 1 in 5 reported a previous history of tuberculosis. As for latent tuberculosis infection, only 58% were tested: 43% tested negative, 15% tested positive and should have received treatment (but only half did) and the remaining 42% were not tested. In the Cox regression bivariate analysis, the proportional-hazards assumption test identified one variable which didn’t have proportional hazards during the follow up: use of antiretroviral treatment time-dependent, and to address this we stratified the Cox regression model.

**Table 1:** Frequencies and bivariate analysis of the association between demographic, socioeconomic and lifestyle factors with the development of tuberculosis in people living with HIV, Recife, 2010.

	N (%)	HR (CI 95%)	*p*	Test of proportional-hazards assumption (*p)*
**Demographic variables**				
**Sex**				
Female	809 (39.1)	1.0		
Male	1260 (60.9)	1.53 (1.07–2.17)	0.019	0.4478
**Age group (years)**				
18–39	1062 (51.3)	1.0		
≥40	1007 (48.7)	0.89 (0.65–1.23)	0.496	0.2776
**Socioeconomic variables**				
**Social support**				
Living with family	1667 (80.8)	1.0		
Living alone or in shelters	395 (19.2)	0.95 (0.63–1.44)	0.817	0.7090
**City of residence**				0.1893
Recife	841 (40.9)	1.0		
Metropolitan region	916 (44.6)	0.81 (0.57–1.15)	0.244	0.0710
Other cities	298 (14.5)	0.85 (0.51–1.40)	0.515	0.3988
**Literacy**				
Yes	1832 (88.8)	1.0		
No	232 (11.2)	1.95 (1.29–2.94)	0.001	0.5928
**Head of the family monthly income**				
≥1 minimum wage	1378 (68.7)	1.0		
<1 minimum wage	627 (31.3)	1.28 (0.90–1.81)	0.168	0.7822
**Variables related to lifestyle**				
**Alchohol consumption**				
None or light drinker	1832 (88.6)	1.0		
Heavy drinker	235 (11.4)	0.94 (0.56–1.57)	0.805	0.5791
**Smoking status**				0.3675
Never	933 (45.1)	1.0		
Former	537 (25.9)	1.17 (0.77–1.77)	0.446	0.9909
Current	599 (29.0)	1.53 (1.05–2.23)	0.026	0.2029
**Smoking status**				
Never or former	1470 (71.0)	1,0		
Current	599 (29.0)	1.44 (1.03–2.02)	0.034	0.1563
**Recreational drugs use**				
Never	1501 (72.6)	1.0		
Past or current	568 (27.4)	1.82 (1.31–2.53)	0.000	0.0968

**Table 2:** Frequencies and bivariate analysis of the association between clinical, laboratory and treatment HIV-related factors, tuberculosis and health services factors with the development of tuberculosis in people living with HIV, Recife, 2010.

	N (%)	HR (CI 95%)	*p*	Test of proportional-hazards assumption (*p)*
**Clinical, laboratory and treatment HIV-related variables**				
**General health variables**				
**BMI**				
≥18.5 kg/m^2^	1869 (92.5)	1.0		
<18.5 kg/m^2^	151 (7,5)	2.87 (1.87–4.42)	0.000	0.7917
**Anemia**				0.6060
No	954 (46.1)	1.0		
Yes	642 (31.0)	3.75 (2.51–5.62)	0.000	0.3260
Missing	473 (22.9)	2.41 (1.50–3.87)	0.000	0.6671
**HIV related variables**				
**Antirretroviral treatment (baseline)**				
Yes	1635 (79.3)	1.0		
No	428 (20.7)	1.13 (0.76–1.67)	0.545	0.0130
**Antirretroviral treatment (time-dependent)**				
Yes	–	1.0		
No	–	1.42 (0.95–2.13)	0.086	0.0216
**CD4 cell count (baseline)**				
≥200 cel/mm^3^	1430 (81.7)	1.0		
**<**200 cel/mm^3^	321 (18.3)	4.50 (3.18–6.37)	0.000	0.5379
**Tuberculosis and Health Services variables**				
**Previous tuberculosis treatment**				
No	1582 (78.3)	1.0		
Yes	439 (21.7)	2.45 (1.75–3.44)	0.000	0.9172
**Indication for LTBI treatment**				0.4595
No indication and no treatment	888 (42.9)	1.0		–
Indication and treatment	153 (7.4)	0.60 (0.21–1.70)	0.340	0.9233
Indication and no treatment	152 (7.4)	5.81 (3.65–9.23)	0.000	0.1145
Tuberculin test not performed	876 (42.3)	1.99 (1.34–2.95)	0.001	0.4950

Comparing those who developed incident tuberculosis and those who did not, there were statistically significant differences in the average monthly income of the head of household (456.3 and 661.9 reais); average BMI (21.4 and 23.9 Kg/m^2^); and average baseline CD4 count (275.2 and 456.7 cel/mm^3^) (data not shown).

Table 3 shows the final Cox multivariate regression model for the association between exposures and incidence of TB stratified by use of HAART (time-dependent). Remained in the model, by significantly increasing the risk of TB: indication for but non-implementation of treatment for LTBI and TST not tested; baseline CD4 cell count <200 cell/mm^3^; anemia (and not testing for it); previous treatment for TB; BMI <18.5 kg/m^2^ and being illiterate. The proportional hazards test was respected in the multivariate model (p = 0.8294). Table 4 shows that among the subgroup with a history of TB treatment, when compared to those with a TST<5 mm, patients with a TST≥5 mm who didn’t receive preventive treatment had a higher risk of TB (HR = 6.70) than patients with TST≥5 mm who receive preventive treatment for TB (HR = 1.67). When we compare only those who tested positive among those with previous treatment, those who did not receive treatment had three times the risk for TB than those who did not receive treatment (HR: 3.36, 95%–CI: 0.37–30.5) though the difference was not statistically significant.

**Table 3:** Cox regression multivariate final model of the association between studied variables and tuberculosis development in people living with HIV, Recife, 2010.

	Hazard ratio adjusted (95% CI)	*p*
**Indication for LTBI treatment**		
No indication and no treatment	1.0	
Indication and treatment	0.74 (0.22–2.44)	0.622
Indication and no treatment	7.90 (4.67–13.37)	0.000
Tuberculinic test not performed	1.63 (1.04–2.54)	0.032
**CD4 cell count (baseline)**		
≥200 cel/mm^3^	1.0	
**<**200 cel/mm^3^	4.10 (2.77–6.08)	0.000
**BMI**		
≥18.5 kg/m^2^	1.0	
<18.5 kg/m^2^	2.25 (1.38–3.67)	0.001
**Anemia**		
No	1.0	
Yes	2.93 (1.86–4.62)	0.000
Missing	2.07 (1.17–3.67)	0.012
**Previous tuberculosis treatment**		
No	1.0	
Yes	2.42 (1.65–3.55)	0.000
**Literacy**		
Yes	1.0	
No	1.74 (1.09–2.80)	0.021

Stratified by antiretroviral treatment.

Test of proportional-hazards assumption (p = 0.8294).

**Table 4:** Cox regression multivariate model of the association between Indication for LTBI treatment and tuberculosis development according to previous tuberculosis treatment in people living with HIV, Recife, 2010.

	No Previous tuberculosistreatment		Previous tuberculosistreatment	
	Hazard ratio Adjusted* (95% CI)	*P value*	Hazard ratio Adjusted* (95% CI)	*P value*
**Indication for LTBI treatment**				
No indication and no treatment	1.0		1.0	
Indication and treatment	0.59 (0.14–2.51)	0.473	1.67 (0.20–13.9)	0.637
Indication and no treatment	9.04 (4.95–16.5)	0.000	6.70 (2.15–20.8)	0.001
Tuberculinic test not performed	1.43 (0.81–2.51)	0.214	2.13 (0.93–4.85)	0.073

* Adjusted for CD4 cell count, Antirretroviral treatment, BMI, Anemia and literacy.

Smoking did not remain in the final model. When we examined the risk of tuberculosis associated with smoking separately for PLHIV according reported use of drugs, there was no increase in risk of TB associated with smoking in those who did not use drugs, and a doubling of the risk of tuberculosis with smoking (current and past) in those who reported using drugs, but this did not reach statistical significance. Use of drugs was more frequent in ex-smokers than in never smokers and in current smokers than in ex-smokers (data not shown).

## Discussion

The incidence rate of tuberculosis was 2.8 per 100 person-years. Risk of TB was higher in those with markers of TB infection (positive TST but no treatment of LTBI, and TB treatment in the past); those with inadequate control of HIV infection (low CD4 count; non-use of HAART), those with poor general health (low BMI; anemia) and illiterate. The probability of survival free of tuberculosis after an individual average 2.5 years of follow up was 91%. This is consistent with a study in Brazil which followed 281 patients with 13 incident cases in 60 months [15] and higher than a study in South Africa which followed 346 with 27 incident cases in 5 years [16]. This may have resulted from good access to free at the point of use supply of antiretroviral treatment in Brazil, which has been shown to be a protective factor for TB [17], [18]. 79.3% of patients in our study reported use of HAART on entry into the cohort. The incidence rate of 2.8 per 100 person/years is intermediate between those in Africa [19]–[21], and those in developed countries [22], [23] and parts of Asia [18]. A much smaller Brazilian study [15], found a lower rate; there were no other Brazilian studies.

In addition to the often studied clinical and laboratory factors, we also investigated whether socioeconomic and lifestyle factors are associated with higher risk of TB in PLHIV. Although average income of individuals with incident TB was lower than in individuals without TB (p = 0.000), only illiteracy of the socio economic factors studies remained in the final model, doubling the risk of developing TB. Poverty and illiteracy were closely associated [24]. The main lifestyle variables in this analysis were smoking and use of recreational drugs. Smoking has not been sufficiently studied as a risk factor for incident tuberculosis in PLHIV, although there is clear evidence of an effect of smoking in the general population: a meta-analysis of this association in HIV negative people found that current smokers were 2.6 and former smokers 1.6 more likely to develop pulmonary TB when compared with never smokers [25], and smoking was associated with higher risk of progression from LTBI infection to disease [26], of recurrence of TB [27] and of mortality from TB [28].Smoking is often more common in PLHIV than in the general population [29]. Miguez-Burbano and colleagues [30] in the United States reported a doubling of risk of development of TB in PLHIV who smoked for 20 years or more.

In our study smoking did not remain independently associated with incident TB in multivariate analysis in spite of the fact that remarkably high prevalences of reported past (25.9%) and current (29.0%) smoking were found. The absence of an effect of smoking was unexpected.

The use of recreational drugs, though statistically significant in the bivariate analysis, was excluded from the multivariate model due to the absence of proportionality in this step, limiting the investigation of its effect on incident tuberculosis.

In the literature it has been reported that recreational drugs users are at a higher risk of TB disease independently of HIV status [5], [31]. TB outbreaks among non-injecting drugs users have been identified and thought to be due both a consequence of sharing drug equipment and poor ventilation [31], [32].

Tuberculosis and HIV infection are known to be associated with malnutrition [33], although the picture is more complex, as with the prevalence of overweight/obesity increases among PLHIV receiving HAART [34]. Even so body mass index <18.5 kg/m^2^, has been reported to be associated with TB in PLHIV [35] and this was consistent with our findings. Anemia, as well as low BMI, is common in chronic infections and can reach levels of up to 95% prevalence in individuals infected with HIV, and is also is associated with progression to AIDS and increased risk of death [36]. In our study 22.9% of patients were not tested for anemia, but of those who were, 40% were anemic, and both having anemia and not having a test were associated with TB, consistently with the literature [37]. In patients diagnosed with tuberculosis in Korea, most of the anemia (64.5%) reverted during or after therapy for TB [38].

The use of HAART is associated with a marked increase in survival of PLHIV, as it improves immune condition and decreases risk of opportunistic infection: HAART has been reported to reduce the proportion of people with HIV that develop TB by more than 80% [19] in spite of the fact that PLHIV receiving HAART have a longer life expectancy (and therefore a longer number of years at risk of developing tuberculosis [18]. A recent systematic review and meta-analysis of the impact of HAART on incidence of tuberculosis in PLHIV in developing countries show a reduction in TB incidence across all baseline CD4 counts [39]. In our study, the non-use of HAART was associated with an increased risk of incident TB (HR = 2.9), consistent with the literature [13], [19], [20], [22].

Having a low CD4 count increased the incidence of TB by nine fold in our study. This was higher than in most studies that looked at effect of baseline CD4 count [16], [22], [40], but similar to that found by Lawn et al [17], who, similar to our study, took into account variations in CD4 count with time and therefore estimated the effect of the CD4 count at the nearest time before the TB occurrence.

There is consensus in the literature that the treatment of LTBI reduces the risk of active TB in PLHIV [41]–[43]; this is the reason for the WHO recommendation for testing and isoniazid preventive therapy (IPT) for a minimum of six months [44]. In our study, patients who had TST≥5 mm but who did not receive treatment had an almost tenfold increase in risk of incident TB (HR = 9.8); whereas those treated had no increase in risk. Starting treatment of TBLI for HIV infected patients in routine in Brazil remains the decision of the attending physician [42], [45]; in our study only half of those identified with LTBI were treated. Equally concerning, 42% of all PLHIV in our study were not TST tested, contrary to WHO [46] and the Ministry of Health in Brazil [8] recommendations. Treatment of LTBI is not simple: it requires two visits for testing (purified protein derivative inoculation and reading), prescription of treatment and patient compliance with additional medication, but evidence is overwhelming that it reduces the risk of TB in PLHIV [13], [47].

Thirty-eight percent of incident TB cases in our study reported TB treatment in the past, a higher percentage than those found in parts of Asia (22%) and the Ivory Coast (24%) [18], [48]. Although TB recurrence occurs more frequently in PLHIV [49] due to immune depletion and consequent failure to contain the bacillus [50], very few epidemiological studies included a history of past TB in the risk factors model for incident TB in PLHIV: Seyler et al [48] found an association and Lawn et al [16] found no association; until recently there were no specific recommendations for PLHIV with a history of TB. In our study, we found a doubling of risk in those with a history of TB; although, among those with previous tuberculosis treatment, there was a 3 fold increase in risk of TB for those who are TST positive and did not receive a new treatment after the TST when compared to those who received a new treatment, this result did not reach statistical significance at the 95% level, and requires further investigation. As in our study the indication of the LTBI treatment was made by the assistant physician, based on clinical judgment, indication bias cannot be excluded. Only a randomized controlled trial could overcome this limitation. A recent WHO guideline strongly recommend IPT for at least six months, for those who are unlikely to have active TB, including patients who had a previous TB treatment [51].

Our study had some limitations. This was an operational research, following patients in a routine setting, so entry into the follow up was not triggered by a common clinical criterion. This is the main limitations of this study: the fact that risk onset is an arbitrary point in time (date of recruitment), and not related to any health/risk related event and therefore the sample is not homogeneous in relation to several factors; so that there may be some bias in the analysis of variables associated to the risk of TB. Most tests and all interventions reflected the decision of the clinician responsible for the routine treatment of these patients, although additional data collection, including record reviews and questionnaires, were initiated by the study. As a consequence, diagnosis of TB was done by the attending physician, whether bacteriologically confirmed or not. We are confident we included all diagnosed cases of TB (because we searched in SINAN and TB treatment is only released for patient registered in SINAN). We exclude TB cases diagnosed within one month of entry into the cohort to ensure that our cohort were tuberculosis free at the start of the follow. To minimize misclassification in relation to loss of follow-up and death, searches were conducted in the SIM-PE [9]. Our study had some specific strengths. The cohort of HIV infected people was followed prospectively, allowing identification of incident cases. We used HAART as time-dependent variables, taking into account the time that each patient was at risk and reflecting closely changes in use of antiretroviral therapy during the follow-up. Including socioeconomic and lifestyle habits as variables in the study allowed us to identify that the risk of incident TB was higher among those illiterate (the well-known association between poverty and tuberculosis continuing in PLHIV).

### Conclusions

Our findings identify some action that can have a substantive impact in reducing tuberculosis both at the individual and in public health level. The findings of the increase in incident TB in PLHIV drug users (and tobacco, although not significantly) are new and offer the possibility of targeted interventions. The interaction between tuberculosis and HIV control programs is critical to the success of prevention of TB, and this should be articulated in reference health services for HIV-infected individuals.
